# Editorial: Nano-(Bio)Catalysis in Lignocellulosic Biomass Valorization

**DOI:** 10.3389/fchem.2018.00577

**Published:** 2018-11-27

**Authors:** Rafael Luque, Christophe Len, Konstantinos Triantafyllidis

**Affiliations:** ^1^Departamento de Química Orgánica, Universidad de Córdoba, Córdoba, Spain; ^2^Peoples' Friendship University of Russia (RUDN University), Moscow, Russia; ^3^Chimie ParisTech, CNRS, Institut de Recherche de Chimie Paris, PSL Research University, Paris, France; ^4^Centre de Recherche Royallieu, Universitéde Technologie de Compiègne (Sorbonne Universités), Compiégne, France; ^5^Department of Chemistry, Aristotle University of Thessaloniki, Thessaloniki, Greece

**Keywords:** lignocellulosic biomass, (bio)catalysis, biofuels, platform chemicals, added value products

The valorization of lignocellulosic biomass, in the form of forest and agricultural wastes, industrial processing side-streams, and dedicated energy crops, toward chemicals, fuels and added-value products has become a major research area with increasing exploitation potential (Lange, 2007; Zhou et al., 2011; Tuck et al., 2012). The efficient and tailored depolymerization of biomass or its primary structural components (hemicellulose, cellulose, and lignin) to platform chemicals with varying functionalities, i.e., sugars, phenolics, furans, ketones, organic acids, etc. is highly dependent on the development of novel or modified chemo- and bio-catalytic processes that take into account the peculiarities and recalcitrance of biomass as feedstock, compared for example to petroleum fractions (Gallezot, 2008; Serrano-Ruiz et al., 2011). In many cases, in order to reach the final product, a series of catalytic reactions/process should be applied in a cascade or “one-pot” mode. A representative example is the bio-based polymer PEF (polyethylene furanoate) which can replace petroleum-derived PET (polyethylene terephthalate) for the production of plastics, where the following sequence of reactions should occur: hydrolysis of cellulose to glucose, isomerization of glucose to fructose, dehydration of fructose to hydroxymethylfurfural (HMF), oxidation of HMF to 2,5-furandicarboxylic acid (FDCA), poly-condensation with ethylene glycol (Avantium^1^).

The most important reactions in biomass conversion include hydrolysis, isomerization, dehydration, hydrogenation, hydrodeoxygenation, hydrogenolysis, oxidation, esterification, ketonization, condensation, and others. Different types of homogeneous and heterogeneous catalysts, as well as biocatalysts (enzymes), with single or dual functionalities (i.e., acidic and hydrogenating, as for example in the acidic zeolite supported Pd, Pt, Ni, etc. catalysts) have been reported so far in the literature, ranging from fundamental catalyst design, to combined optimization of catalyst properties and reaction conditions, to pilot scale validation (Serrano-Ruiz et al., 2011; Zhou et al., 2011; Triantafyllidis et al., 2013). Photocatalysis, as well as alternative energy sources (plasma, microwave, and ultrasound) have also been explored for the catalytic conversion of biomass and its derivatives (Colmenares and Luque, 2014; Granone et al., 2018).

Based on these premises, the present Special Issue in *Frontiers in Chemistry, Section of Green and Sustainable Chemistry*, entitled “Nano-(bio)catalysis in lignocellulosic biomass valorization” aims to further contribute to the momentum of research and development in the (bio)catalytic conversion of biomass, by featuring original research papers as well as two review papers, authored and reviewed by experts in the field.

The first review paper provided a comprehensive overview of the recent research on chemical oxidative techniques for the pretreatment of lignocellulosics with the explicit aim to rationalize the objectives of the biomass pretreatment step and the problems associated with the conventional processes (Den et al.). The mechanisms of reaction pathways, selectivity and efficiency of end-products obtained using greener processes such as ozonolysis, photocatalysis, oxidative catalysis, electrochemical oxidation, and Fenton or Fenton-like reactions, as applied to depolymerization of lignocellulosic biomass were summarized with deliberation on future prospects of biorefineries with greener pretreatment processes in the context of the life cycle assessment.

The second review paper discussed the hydrolysis of hemicellulose and the recent advances in the production of furfural (Delbecq et al.). More specifically, the review discussed advances obtained in major production pathways recently explored, splitting them in the following categories: (i) non-catalytic routes, like use of critical solvents or hot water pretreatment, (ii) use of various homogeneous catalysts like mineral or organic acids, metal salts, or ionic liquids, (iii) feedstock dehydration making use of various solid acid catalysts; (iv) feedstock dehydration making use of supported catalysts, (v) other heterogeneous catalytic routes. The paper also briefly overviewed current understanding of furfural chemical synthesis and its underpinning mechanism.

The topic of efficient and controlled cellulose hydrolysis, not using enzymes or strong inorganic acids, has attracted considerable interest. To this end, one of the original research papers of this Special Issue focused on the mechanocatalytic depolymerization of cellulose with perfluorinated sulfonic acid ionomers (Karam et al.). Very high yields of water soluble sugars (90–97%), mostly as oligosaccharides with a degree of polymerization (DP) up to 11, were obtained under optimized conditions using Aquivion PW98 and PW66, respectively, as solid acid catalysts.

Although, homogeneous and heterogeneous chemo-catalysis can offer high reaction rates as well as other benefits, enzymatic catalysis provides exceptionally high selectivities to the desired products. In a related paper of this issue, it was shown how the enzymatic hydrolysis of organosolv pretreated forest materials can be fine-tuned toward the efficient production of cellobiose, a non-digestible oligosaccharide (NDO), which together with other cello-oligosacharides (COS), are considered as prebiotic candidates that have been related to the prevention of intestinal infections and other disorders for both humans and animals (Karnaouri et al.). In this work, the heterologous expression and characterization of two Cellobiohydrolases (CBHs) from the filamentous fungus Thermothelomyces thermophila, and their synergism with endoglucanases (EGs) for cellobiose release from organosolv pretreated spruce and birch, was systematically studied and discussed.

Levulinic acid, being produced from HMF, has been recognized as a very important platform chemical that can be catalytically converted further to a series of functional chemicals. In the present special issue, two papers have focused on the hydrogenation of levulinic acid toward γ-Valerolactone, another very important chemical with a wide range of uses. The first paper deals with the development of silylated zeolitic catalysts (3 wt.% Pt on zeolite Y) with enhanced hydrothermal stability for the aqueous-phase hydrogenation of levulinic acid to γ-Valerolactone (GVL) (Vu et al.). It was shown that by the use of trichlorosilanes as silylating agents, the hydrothermal stability of zeolite Y can be improved significantly, although an inhibition in the yield of GVL was observed due to blockage of the pores by the silane. The second relevant paper presents a study of nickel modified zeolite optimization as bi-functional catalyst (Ni/HZSM-5) for the vapor-phase hydrogenation of levulinic acid to GVL (Popova et al.). The content and state of nickel and its interaction with the zeolite were critical parameters affecting both the acidity of the zeolite as well as the reducibility of Ni. At the best case, the authors reported 99% conversion of levulinic acid and 100% selectivity to GVL at 320°C.

Aldol condensation and C-C coupling reactions in general are also very important in biomass valorization, especially when the targeted products are hydrocarbon based transportation fuels, such as gasoline and diesel. In a relevant paper of this issue, the physico-chemical properties of MgGa mixed oxides derived from the corresponding Layered Double Hydroxides (LDHs), as well as of their reconstructed layered analogs, were studied and correlated to their performance in aldol condensation of furfural and acetone (Kikhtyanin et al.). It was shown that the basicity and the textural properties of the MgGa materials determined their catalytic activity and selectivity while their properties resembled those of MgAl hydrotalcite-based materials.

Fast pyrolysis is one of the most promising thermochemical processes for the direct conversion of biomass into a liquid product, the so called pyrolysis oil or biooil, with gases and char being formed to a lesser extent. Biooil contains water and its organic fraction consists of phenolics, ketones, aldehydes, acids, sugars, and other compounds in minor amounts. It is relatively acidic, unstable and not miscible with petroleum fractions, and needs to be upgraded, usually via (hydro)deoxygenation. One of the papers in this issue, describes the continuous hydrodeoxygenation (HDO) of biooil with an *in situ* sulfided metal oxide catalyst and the effect of reaction temperature (350, 375, and 400°C) on the deoxygenation activity (Treusch et al.).

Lignin has gained increased interest within the biorefinery concept as it can act as source of high added value phenolics and aromatics. It can be depolymerized via various types of hydrothermal processes, such as reductive or oxidative hydrogenolysis, or by fast pyrolysis which produces a biooil that contains essentially various alkoxy-phenols. In a relevant paper, the fast pyrolysis of a kraft lignin (byproduct of the production of cellulose pulp from biomass via the Kraft processes) was compared to the catalytic fast pyrolysis using conventional, mesoporous, and nanosized ZSM-5 zeolite, aiming at the production of a partially deoxygenated biooil, enriched in alkyl-phenols and aromatics (BTX and naphthalenes) (Lazaridis et al.). The effect of ZSM-5's acidity and hierarchical porosity on product yields, selectivity of various compounds and resistance to coking was studied and discussed. In another paper of this issue, monolignols such as sinapyl (SA) and coniferyl (CA) alcohols were linked together with caffeic acid (CafAc) via enzymatic catalysis affording a polymeric network similar with natural lignin (Ion et al.). The production of the valuable compound vanillin from isoeugenol under mild conditions was investigated in another paper included in this special issue, using bifunctional nanocatalysts (iron on sulphonated SBA-15) which were prepared by an alternative mechanochemical method (Ostovar et al.).

In summary, the present special issue addresses various representative reactions and processes in biomass valorization, highlighting the importance of developing novel, efficient and stable nano-(bio)catalysts with tailored properties according to the nature of the reactant/feedstock and the targeted products.

## Author contributions

All authors listed have made a substantial, direct and intellectual contribution to the work, and approved it for publication.

### Conflict of interest statement

The authors declare that the research was conducted in the absence of any commercial or financial relationships that could be construed as a potential conflict of interest.

## Footnotes

^1^Avantium. Available online at: https://www.avantium.com/yxy/yxy-technology/
